# Supplementary figures and images for: Empagliflozin Prevent High-Glucose Stimulation Inducing Apoptosis and Mitochondria Fragmentation in H9C2 Cells through the Calcium-Dependent Activation Extracellular Signal-Regulated Kinase 1/2 Pathway
Source: Int J Mol Sci. 2024 Jul 28;25(15):8235. doi: 10.3390/ijms25158235 (PMC11311311; doi:10.3390/ijms25158235)

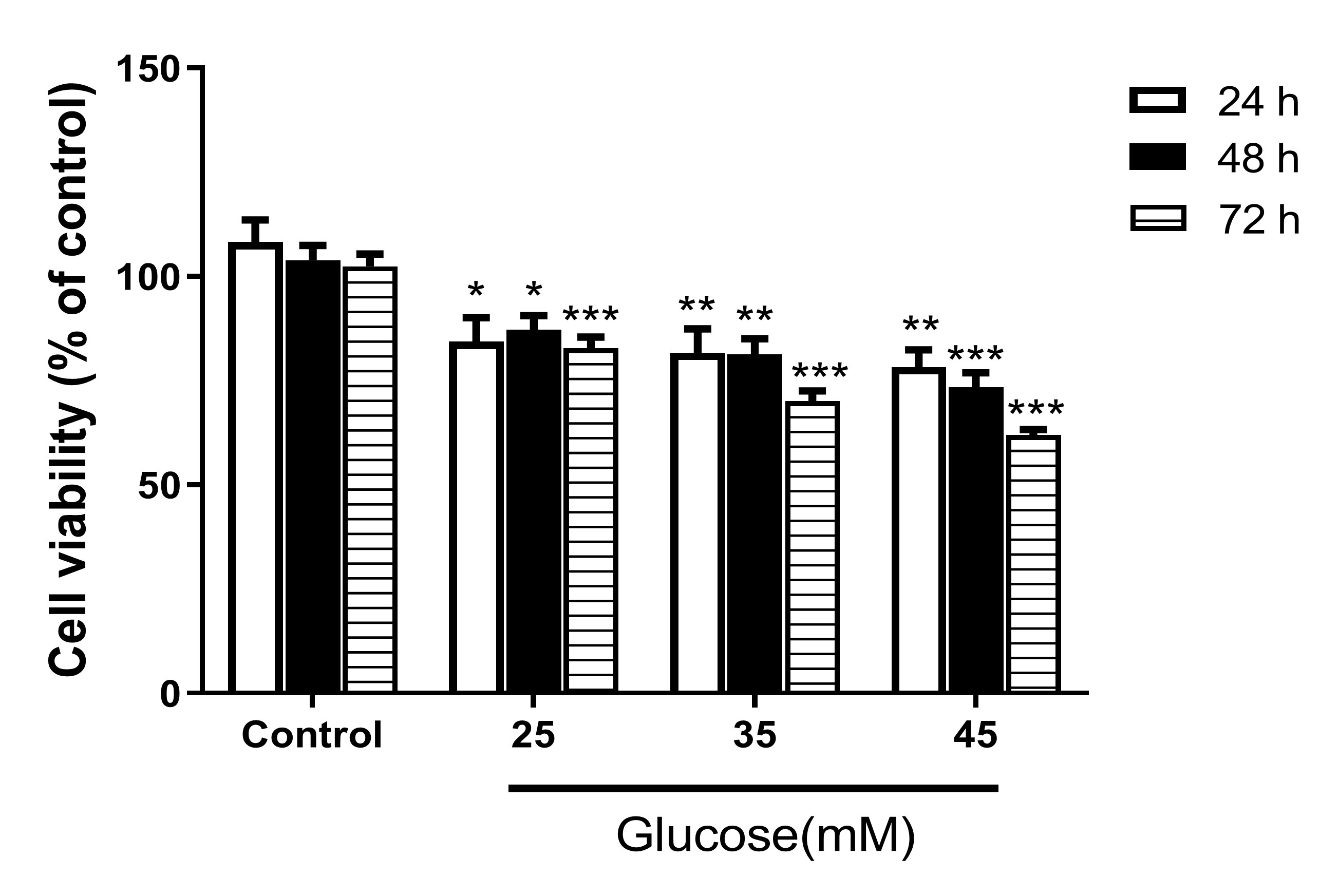

Supplement: Supplementary file 1 [file ijms-25-08235-s001.zip › ijms-3109834-supplementary.jpg]
